# Preoperative Fasting Practices Across Three Anesthesia Societies: Survey of Practitioners

**DOI:** 10.2196/15905

**Published:** 2020-01-28

**Authors:** Richard Neville Merchant, Navraj Chima, Olle Ljungqvist, Juliana Nai Jia Kok

**Affiliations:** 1 Department of Anesthesiology and Perioperative Medicine Royal Columbian Hospital, Fraser Health Authority University of British Columbia New Westminster, BC Canada; 2 Vancouver Coastal Health Authority University of British Columbia Vancouver, BC Canada; 3 Department of Surgery Faculty of Medicine and Health Orebro University Orebro Sweden; 4 Department of Anaesthesia and Pain Management Royal Melbourne Hospital Melbourne Australia

**Keywords:** preoperative care, guideline adherence, practice guideline

## Abstract

**Background:**

Pulmonary aspiration of gastric contents is recognized as a complication of anesthesia. To minimize that risk, anesthesiologists advised fasting for solid foods and liquids for an often prolonged period of time. However, 30 years ago, evidence was promulgated that fasting for clear liquids was unnecessary to ensure an empty stomach. Despite a strong evidence base and the knowledge that fasting may be physiologically harmful and unpleasant for patients, the adoption of society guidelines recommending short fasting periods for clear fluids into clinical practice is uncertain.

**Objective:**

This study aimed to determine the current practices of anesthetists with respect to fasting guidelines.

**Methods:**

An electronic internet survey was distributed to anesthetists in Canada (CAN), Australia and New Zealand (ANZ), and Europe (EUR) during April 2014 to February 2015. The anesthetists were asked about fasting guidelines, their recommendations to patients for the consumption of clear fluids and solid foods, and the reasons and consequences if these guidelines were not followed.

**Results:**

A total of 971 anesthetists completed the survey (CAN, n=679; ANZ, n=185; and EUR, n=107). Although 85.0% (818/962) of these participants claimed that their advice to patients followed current society guidelines, approximately 50.4% (476/945) enforced strict fasting and did not allow clear fluids after midnight. The primary reasons given were with regard to problems with a variable operating room schedule (255/476, 53.6%) and safety issues surrounding the implementation of clear fluid drinking guidelines (182/476, 38.2%).

**Conclusions:**

Many anesthetists continue to follow outdated practices. The current interest in further liberalizing preoperative fluid intake will require more change in anesthesia culture.

## Introduction

A prescription for fasting before surgery is a topic that has been discussed in the literature since modern anesthesia started in 1847. In 1858, John Snow proposed that fasting would be helpful to avoid the unpleasantness of vomiting associated with anesthesia [1]. Following descriptions of regurgitation and pulmonary aspiration of gastric contents [2] later in the 19th century, it was proposed that fasting would help decrease the risk of such complications [3]. Seminal work by Maltby et al [4] in the 1980s and 1990s demonstrated that clear fluids are cleared from the stomach within 2 to 3 hours, thus negating the need for long periods of fasting. Recent evidence suggests that starvation—*nil per os (NPO, nothing by mouth) from midnight*—for clear liquids is not only unnecessary to allow for gastric emptying but could also have deleterious effects in the perioperative period [5].

In the 1990s, evidence and discussions led to proposals that the fasting-from-midnight dogma must be modified and liberalized. Maltby [6] lists 91 key references in summarizing the debate and the literature. The recommendations for liberal perioperative fasting guidelines in elective patients have gradually been adopted by numerous national societies, including the Canadian Anesthesiologists’ Society, whose guidelines were modified in 1998 to include these liberal fasting policies (further changes were made in 2015 to include recommendations encouraging the consumption of clear fluids preoperatively) [7]. In Norway, similar guidelines were published in 1994 (further updated in 2005) [8], and the American Society of Anesthesiologists did so in 1999 [9]. With the development and implementation of many *enhanced recovery after surgery* protocols, which emphasize preoperative preparation, there is renewed interest in shortening the fasting period for clear fluids to less than that recommended by these guidelines, particularly in pediatric anesthesia. [10].

Despite the extensive knowledge base developed and the dissemination of society guidelines based upon the published science, literature suggests that adoption into clinical practice has been irregular. In Canada (CAN), many hospital departments appear to have policies unchanged from the traditional *NPO from midnight*, although it has been 20 years since the society guidelines were changed. Dr Maltby himself recently experienced a further variation of the reality of our imperfect system—he was allowed fluids in preparation for an elective operation; however, he then subsequently fasted for 20 hours when his elective procedure was *bumped* from the schedule in a major Canadian academic teaching hospital.

To review the current practices in preoperative fasting advice, we conducted an electronic survey on anesthetists from CAN, Australia and New Zealand (ANZ), and Europe (EUR). We wished to determine the current practices and perceptions surrounding fasting guidelines, whether the current guidelines are being followed and what could be preventing the uptake of these evidence-based guidelines.

## Methods

### Study Design

The study design and approval from the Research Ethics Board (FHREB number 2014-02) were obtained from the Fraser Health Authority (British Columbia, CAN, on March 12, 2014; chairman Dr Stephen Pierce).

A Web-based survey was designed and sent to anesthetists in 3 major practice regions: in CAN via provincial associations in the largest provinces, in ANZ through the Australian and New Zealand College of Anesthetists to a sample of fellows in accordance with their survey policy, and in EUR to the members of the European Society of Anesthesiology. This was a *convenience sample* of practices thought likely to be similar in the implementation of current *liberal* fasting guidelines based on recent literature. Before designing the survey, we reviewed the current fasting guidelines for the society in each region (see Table 1). Using these recommendations, a series of questions was developed by the authors and advisors (RM and RNM) to explore the currently *prescribed* preoperative fasting advice as well as the features about preoperative fasting such as the source of advice and why society guidelines might not be followed. A common thought is that variability in the time of access to the operating room (OR) will affect the actual fasting time; hence, we also asked whether and how often operations are actually moved earlier than the planned time.

The questionnaire was tested with 20 Canadian anesthesia trainees. It was implemented as a Web-based survey using FluidSurveys (now SurveyMonkey) for the CAN and ANZ participants and separately in SurveyMonkey for the EUR participants.

The survey included 13 questions on fasting experience and practices, each on a separate page, along with a collection of basic demographic and practice information. There were no mandatory questions and no completeness check. All the survey questions can be found in Multimedia Appendix 1.

**Table 1:** Current society guidelines.

Guidelines	Canada [11]	Australia and New Zealand [12]	Europe [13]
Minimum duration of fasting for meat, fried foods, or fatty foods	8 hours	No comment	All solid foods for 6 hours
Minimum duration of fasting for light meal or infant formula	6 hours	6 hours	All solid foods for 6 hours
Minimum duration of fasting for breast milk	4 hours	6 hours	4 hours
Minimum duration of fasting for clear fluids	2 hours	2 hours	2 hours
Active encouragement of clear fluid intake	Yes	No comment	Yes
Use of carbohydrate-rich beverages	No comment	No comment	Yes
Pharmacological intervention	No comment	Consider	Not routine
Gum chewing	No comment	No indication to cancel	No indication to cancel

### Participants

The survey information and invitations were distributed to the anesthesiologists via email with the assistance of provincial anesthesia associations in CAN, the Australian and New Zealand College of Anesthetists, and the European Society of Anesthesiology. This occurred from April to May 2014 for CAN, from January to February 2015 for ANZ, and from December 2014 to August 2015 for EUR.

The invitation email explained the background and the aim of the survey, its ethical approval, the type of demographic and practice information that would be requested, that it was voluntary, that participation would imply consent, and that the responses would remain anonymous. The email included a URL to the survey, which was implemented as an open Web survey.

We did not track the number of invitations sent or whether individual anesthetists did or did not respond to the survey request, and no follow-up or reminder emails were sent.

### Data Analysis

The extracted survey data were collated and analyzed in Microsoft Excel 2016 (Microsoft). All responses to each question were included in the analysis, including those from incomplete surveys. The data have been presented using descriptive statistics, with the number and percentage of respondents in each region. We have mainly reported data for the 3 practice regions separately but have not applied comparative statistics.

## Results

A total of 1057 anesthetists participated in the survey, of which 971 completed the survey questions: CAN (n=679); ANZ (n=185); and EUR (n=107; Table 2).

Overall, in response to the simple question “Do your fasting instructions follow Society guidelines?,” 85% of anesthetists claimed that their advice to patients followed current society guidelines: 84.6% (571/675) in CAN, 88.4% (160/181) in ANZ, and 82.1% (87/106) in EUR (Table 3). However, preoperative fluids after midnight were encouraged by only 45.5% (300/659) CAN anesthetists, 64.1% (116/181) ANZ anesthetists, and 50.0% (53/106) EUR anesthetists. The most common reasons reported for either enforcing fasting or failing to encourage the intake of clear fluids were because of the perceived problems with a variable OR schedule (CAN 194/359, 54%, ANZ 38/64, 58%, and EUR 23/52, 44%) and safety issues related to the implementation of clear fluid drinking (182/476, 38.2%) of anesthetists across all regions). Overall, 22.9% (211/922) of anesthetists specified a maximum volume of clear fluids to patients, and 20.5% (192/935) reported encouraging a specific preoperative fluid (complex carbohydrate or electrolyte; Table 3).

Patients in ANZ and EUR are routinely allowed to take some solid food on the day of surgery, with 89.5% (162/181) of ANZ and 61.3% (65/106) of EUR patients being allowed to eat a light breakfast 6 to 8 hours before induction of anesthesia compared with only 22.5% (149/662) of CAN patients (Table 3). Only 10% to 14% of anesthetists allowed milk in tea or coffee, and a small proportion prescribed a carbohydrate or an electrolyte drink (20% to 25%) in all regions. The routine use of H_2_-receptor antagonists or proton pump inhibitors in elective nonobstetric patients was low, with the highest use (10/99, 10%) in EUR (Table 3).

Operations being moved earlier than the planned time was reported to happen *frequently* by 17.4% (162/930) of anesthetists, *occasionally* by 60.7% (565/930), and *rarely* by 21.8% (203/930) overall (Table 4). These changes in schedule were deemed to cause problems *frequently* by only 5.0% (44/884) of anesthetists, *occasionally* by 41.0% (363/884) of anesthetists, and *rarely* by 54.0% (477/884) of anesthetists overall (Table 4). Overall, 31.0% (274/886) of respondents across all regions indicated that patients frequently comment on being allowed to drink preoperatively (Table 3; though the question did not specifically quantify whether these were comments on being allowed to, or being restricted from, drinking).

**Table 2:** Origin of respondents.

Country or region	Respondents, n
Canada	713
Australia and New Zealand	191
United Kingdom	16
Germany	13
Italy and Spain	8 each
Belgium and Sweden	6 each
Czech Republic, France, Greece, Netherlands, Poland, Portugal, and Switzerland	5 each
Other	33

**Table 3:** Survey responses by region.

Survey questions			Canada		Australia and New Zealand		Europe		Total	
			n/N (%)		n/N (%)		n/N (%)		n/N (%)	
**Do your fasting instructions encourage drinking clear fluid until 2 or 3 hours before scheduled time of surgery?**										
	Yes		300/659 (45.5)		116/181 (64.1)		53/105 (50.5)		469/945 (49.6)	
	**No**		359/659 (54.5)		65/181 (35.9)		52/105 (49.5)		476/945 (50.4)	
		As we don’t agree with the guidelines	1/359 (0.3)		1/65 (2.0)		2/52 (4.0)		4/476 (0.8)	
		As too many of our patients are at high risk	24/359 (6.7)		4/65 (4.0)		0/52 (0.0)		28/424 (6.6)	
		As we cannot establish a system to implement this safely	135/359 (37.6)		26/65 (40.0)		21/52 (40.0)		182/476 (38.2)	
		As the operating room schedule is too variable	194/359 (54.0)		38/65 (58.0)		23/52 (44.0)		255/476 (53.6)	
		Other reasons	106/359 (29.5)		22/65 (34.0)		6/52 (12.0)		134/476 (28.1)	
**Do your fasting instructions routinely allow some solid food on the day of surgery?**										
	No solid food or milk (except breast milk) after midnight on the night before surgery		503/662 (76.0)		15/181 (8.3)		38/106 (35.8)		556/949 (58.6)	
	Solid food/*light breakfast* allowed until 8 (or 6) hours before surgery		149/662 (22.5)		162/181 (89.5)		65/106 (61.3)		376/949 (39.6)	
	Other, please specify		10/662 (1.5)		18/181 (9.9)		2/106 (1.9)		30/949 (3.2)	
**Do you encourage a specific preoperative fluid (complex carbohydrate or electrolyte) as part of your fasting/drinking policies?**										
	Yes		131/656 (20.0)		36/180 (20.0)		25/99 (25)		192/935 (20.5)	
	No		525/656 (80.0)		144/180 (80.0)		74/99 (75)		743/935 (79.5)	
**Do members of your department prescribe preoperative H_2_-receptor antagonists or proton pump inhibitors in healthy patients undergoing elective surgery (not obstetric patients)?**										
	Routinely		21/645 (3.3)		4/174 (2.3)		10/99 (10)		35/918 (3.8)	
	Only when clinically indicated		624/645 (96.7)		170/174 (97.7)		89/99 (90)		883/918 (96.2)	
**Do patients comment on being allowed to drink on day of surgery?**										
	Frequently		181/624 (29.0)		56/171 (32.7)		39/91 (43)		276/886 (31.1)	
	Rarely		382/624 (61.2)		102/171 (59.6)		42/91 (46)		526/886 (59.4)	
	Never		61/624 (9.8)		13/171 (7.6)		10/91 (11)		84/886 (9.5)	

**Table 4:** Scheduling issues.

Rate of event		Canada, n (%)	Australia and New Zealand, n (%)	Europe, n (%)	Total, n (%)
**Occasionally operations are moved earlier from their slated time. In your hospital is this…**					
	Frequent	115 (18)	26 (15)	21 (21)	162 (17)
	Occasional	414 (63)	107 (60)	107 (44)	565 (61)
	Rare	124 (19)	45 (25)	34 (34)	203 (22)
**Have such changes in operative time been observed to cause problems?**					
	Frequently	27 (4)	11 (6)	11 (12)	44 (5)
	Occasionally	254 (41)	47 (48)	31 (33)	363 (41)
	Rarely	347 (56)	47 (46)	53 (56)	477 (54)

## Discussion

### Principal Findings

Our study is the largest published survey of practicing anesthetists in multiple regions and societies. In this survey of preoperative fasting practices, we established that a substantial proportion of anesthetists responding to our survey across all 3 regions impose strict fasting requirements for both solids and fluids after midnight before surgery. Problems with a variable OR schedule and safety issues related to the implementation of clear fluid drinking guidelines were the most common reasons cited for maintaining these outdated practices.

Starving from midnight seems hardly apt any more given the clear evidence supporting the consumption of clear fluids up until 2 hours before surgery [4,5] for patients not otherwise at risk of delayed gastric emptying. The current fasting guidelines [7-9] have increasingly promoted more liberal advice to the consumption of fluids before surgery. Despite this, it seems that clinical practice in large part lags behind the evidence and society guidelines. Although 85% of anesthetists indicated that they follow the current fasting guidelines, only 54% of anesthetists in CAN and EUR reported allowing the consumption of clear fluids 2 to 3 hours before the surgery.

With regard to solid foods, a greater proportion of ANZ anesthetists allowed some solid food on the day of surgery compared with anesthetists from EUR and especially CAN. Most clinicians in ANZ and EUR allow a light breakfast on the morning of the surgery. This may be because of the difference in the manner of OR scheduling. Many European and Australian OR suites have fixed morning and afternoon lists which are usually not changed and which allow a clear period of fasting after consumption of a meal (in comparison with CAN where OR procedure schedules are typically booked for a whole day). Despite this, the practice of allowing fluids until 2 to 3 hours before surgery is similar among regions at only approximately 50%.

Although the most commonly cited reason for not adhering strictly to the society guidelines was the variability in the OR schedule; 83% of respondents stated that such changes happened only occasionally or rarely. The second most common reason quoted, by 36% of respondents, was that “we cannot establish a system to implement this safely.” Changes to the OR schedule were reported to cause a problem *frequently* in less than 5% of cases, which is somewhat inconsistent with the safety concern. It is certainly the case in the Royal Columbian Hospital in CAN that cases are rarely pushed up so far as to cause a problem with fasting for fluids (RN Merchant, unpublished data).

The risk to which these responses refer is presumably vomiting and pulmonary aspiration of gastric contents, to which John Snow referred in 1858, as quoted by Maltby [6]:

In his 1858 book on chloroform, he again commented on the unpleasantness of vomiting, but not on its danger: ‘chloroform is very apt to cause vomiting, if inhaled while there is a quantity of food in the stomach. The sickness is not attended with any danger but it constitutes an unpleasantness and inconvenience which it is desirable to avoid.’

However, the risk of aspiration is being reexamined by many authorities, particularly in pediatric anesthesia. Beach et al, representing the Pediatric Sedation Research Consortium (PSRC), analyzed the PSRC database and reported an aspiration rate of 8 cases among 82,546 patients who fasted and 2 cases among 25,401 patients who did not fast [14]. Brady et al, in a Cochrane review of 22 studies, found that different fasting regimens were not associated with differences in complications and patients who had consumed fluids preoperatively had the same or smaller residual gastric volume and there was no difference in complications but greater patient satisfaction [15].

Adopting more liberal fasting guidelines will not necessarily translate to equivalent reductions in actual fasting times. Implementation issues include poor quality of available information, especially from internet-based resources [16]; misunderstanding of guidance by the patients (a concern among some of the respondents in our survey); and patients’ own (mis)perceptions that fasting is better. In Brazil, de Aguilar-Nascimento et al in the aptly named BIGFAST study reported that the actual median preoperative fasting time was 12 hours [17]. The fasting time was longer in hospitals using an older fasting protocol than in those that had adopted new guidelines, but 80% of the patients were operated on after 8 or more hours of fasting and 46% after more than 12 hours. Similarly, in South Africa, Lamacraft et al found that the median duration of fasting was 14 hours for solids and over 13 hours for oral fluids, despite fasting guidelines similar to those quoted for our regions [18].

Results such as these are not limited to the developed world. Njoroge et al reported the results of a survey of patients and providers in Kenya that showed that most patients believed they should fast, although only 25% of the providers prescribed a 2-hour fast for fluids [19]. Similarly, Gebremedhn et al reported that although Association of Anesthetists of Great Britain and Ireland guidelines were in place in their Ethiopian hospital, 95% of the patients fasted for fluids for longer, with a mean of 19 hours [20].

Our review did not determine the actual fasting times in our countries, but it is clear that the effort must go beyond promoting guidelines. The adoption of quality improvement methods may provide a useful strategy for changing practice as demonstrated in a recent initiative in a pediatric setting [21]. Indeed, fasting protocols and practices have become a priority debate in the pediatric anesthesia literature [22,23], and it seems likely that more liberal fasting practices may have particular benefit in children [24,25]. One novel approach may be to provide patients with fluids after they are admitted to hospital. Allowing patients to drink until they were transferred to the OR appeared to reduce postoperative nausea and vomiting without apparent evidence of harm [26] and provided further evidence that even a 2-hour fluid fast may be excessively conservative.

### Limitations

There are several limitations to the study. First, our results are based on a self-reporting group of anesthetists and represent a small proportion of the total number of practicing physicians from each region. This relatively small sample size limits our ability to make accurate comments on the differences among regions; however, it still allows us to analyze how a group of anesthetists across the Western world view how they are practicing in the modern era. We sent only 1 invitation to the survey and did not track the respondents or those who failed to respond.

Second, the surveys were distributed at different times because of constraints in the survey distribution. This could have changed how physicians responded because of new literature being presented or the greater adoption of guidelines. However, the society guidelines in each region did not change during our survey distribution, so it is unlikely that these would cause large swings in the anesthesia practice.

Finally, the perception of anesthesiologists may not be the actual advice given to patients as a significant proportion report that the clinic paramedical staff and surgeons give advice. We did not review the advice given by those sources. We have not posted our data on a Web resource.

### Conclusions

Preoperative fasting as a protective maneuver from pulmonary aspiration of gastric contents has been discussed since 1848, but the guidelines developed in the 1950s swayed for many years and, in large part, continue to do so despite the evidence-based research and updated guidelines presented over the last 30 years. A substantial and clinically important number of anesthesiologists from our sample of practitioners from CAN, ANZ, and EUR continue to follow practices that have been replaced by guidelines and standards based on research from the 1980s and 1990s. Many believe that these practices are unpleasant and potentially harmful to patients [10]. The current interest in liberalizing fluid administration even further will require a further change in the culture of anesthesia practice on an ongoing basis.

## Appendix

Multimedia Appendix 1 Survey questions of preoperative fasting practices across 3 anesthesia societies.

Multimedia Appendix 2 Presentation of our work at the IARS Annual Meeting in 2015 in Honolulu Hawaii.

## Abbreviations

ANZ: Australia and New Zealand
CAN: Canada
EUR: Europe
NPO: nil per os
OR: operating room
PSRC: Pediatric Sedation Research Consortium
