# Supplementary material for: Preoperative Fasting Practices Across Three Anesthesia Societies: Survey of Practitioners
Source: JMIR Perioper Med. 2020 Jan 28;3(1):e15905. doi: 10.2196/15905 (PMC7709845; doi:10.2196/15905)
Supplement: Multimedia Appendix 1 [file periop_v3i1e15905_app1.docx]

**A Survey of Pre-Operative Fasting Practices Across 3 Different Anaesthesia Societies**

Navraj Chima, Olle Ljungqvist, Ian Smith, Juliana Kok, Roger Maltby, Richard N Merchant

Survey Questions

**Are you willing to participate in this survey?**

Yes

No

**Who determined preoperative fasting guidelines in your hospital in the 1980's?**

Not known

Anesthesiology

Surgery

Nursing

All of the above

**Who determines preoperative fasting guidelines in your hospital in 2014?**

Head of Anesthesiology

Individual Anesthesiologist

Head of Surgery

Individual Surgeon

Nursing Policy

**Who routinely provides preoperative fasting guidelines to patients your hospital in 2014?**

Preoperative clinic nurse

Anesthesiology

Surgeon or surgeon's office

**Do your fasting instructions follow Society Guidelines?**

Yes

No

**Do your fasting instructions follow ESA guidelines?**

Yes

No

**Do your fasting instructions encourage drinking clear fluid until 2 or 3 hours before scheduled time of surgery?**

Yes

No, because:

We don't agree with the guidelines

too many of our patients are high risk

we cannot establish a system to implement this safely

the OR schedule is too variable

other

**Do your fasting instructions specify a maximum volume of clear liquid?**

Yes

No

**Do your fasting instructions routinely allow some solid food the day of surgery?**

No solid food or milk (except breast milk) after midnight the night before surgery

Solid food / "light breakfast" allowed until 8 (or 6) hours before surgery?

Other, please specify...

**Do your fasting instructions allow a small volume (20-30 ml) of milk in black coffee or tea?**

Yes

No

**Do your encourage a specific preoperative fluid (complex carbohydrate or electrolyte) as part of your fasting/drinking policies?**

Yes

No

**Do members of your department prescribe preoperative H2-receptor antagonists or proton pump inhibitors in healthy patients undergoing elective surgery (not obstetric patients)?**

Routinely

Only when clinically indicated

**How do you achieve agreement among anesthesiologists, surgeons, nurses when changes in policy are required? (please describe)**

Collaborative discussion, decision made by health care staff

No agreement, anesthesia chooses

No agreement, surgeon chooses

No agreement, nurses chooses

No agreement, operative room administrator chooses

Patient chooses

Other

**Occasionally operations are moved earlier from their slated time. In your hospital is this:**

Frequent

Occasional

Rare

**Have such changes in operative time been observed to cause problems?**

Frequent

Occasional

Rare

**Have you ever seen a complication in your elective practice which you can attribute to fasting policies?**

Open-ended response

Do patients comment on being allowed to drink on day of surgery?

Never

Rarely

Frequently

Other

**Country**

**Primary or major hospital/institution of practice:**

**Years in anesthesia practice:**
