# Supplementary material for: Preoperative Fasting Practices Across Three Anesthesia Societies: Survey of Practitioners
Source: JMIR Perioper Med. 2020 Jan 28;3(1):e15905. doi: 10.2196/15905 (PMC7709845; doi:10.2196/15905)
Supplement: Multimedia Appendix 2 [file periop_v3i1e15905_app2.ppt]

## Slide 1
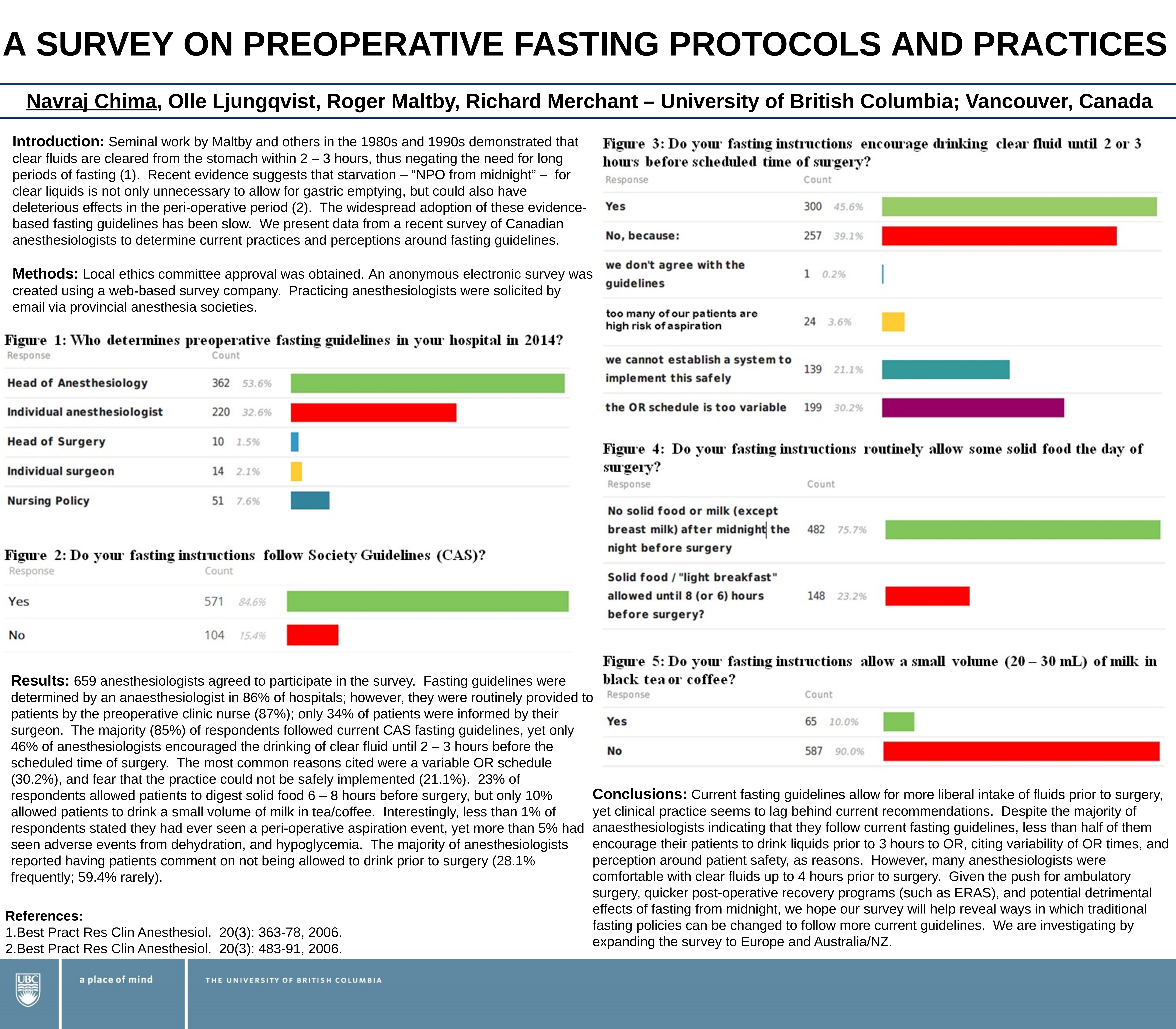

A SURVEY ON PREOPERATIVE FASTING PROTOCOLS AND PRACTICES
Navraj Chima, Olle Ljungqvist, Roger Maltby, Richard Merchant – University of British Columbia; Vancouver, Canada
Introduction: Seminal work by Maltby and others in the 1980s and 1990s demonstrated that clear fluids are cleared from the stomach within 2 – 3 hours, thus negating the need for long periods of fasting (1). Recent evidence suggests that starvation – “NPO from midnight” – for clear liquids is not only unnecessary to allow for gastric emptying, but could also have deleterious effects in the peri-operative period (2). The widespread adoption of these evidence-based fasting guidelines has been slow. We present data from a recent survey of Canadian anesthesiologists to determine current practices and perceptions around fasting guidelines.
Methods: Local ethics committee approval was obtained. An anonymous electronic survey was created using a web-based survey company. Practicing anesthesiologists were solicited by email via provincial anesthesia societies.
Results: 659 anesthesiologists agreed to participate in the survey. Fasting guidelines were determined by an anaesthesiologist in 86% of hospitals; however, they were routinely provided to patients by the preoperative clinic nurse (87%); only 34% of patients were informed by their surgeon. The majority (85%) of respondents followed current CAS fasting guidelines, yet only 46% of anesthesiologists encouraged the drinking of clear fluid until 2 – 3 hours before the scheduled time of surgery. The most common reasons cited were a variable OR schedule (30.2%), and fear that the practice could not be safely implemented (21.1%). 23% of respondents allowed patients to digest solid food 6 – 8 hours before surgery, but only 10% allowed patients to drink a small volume of milk in tea/coffee. Interestingly, less than 1% of respondents stated they had ever seen a peri-operative aspiration event, yet more than 5% had seen adverse events from dehydration, and hypoglycemia. The majority of anesthesiologists reported having patients comment on not being allowed to drink prior to surgery (28.1% frequently; 59.4% rarely).
Conclusions: Current fasting guidelines allow for more liberal intake of fluids prior to surgery, yet clinical practice seems to lag behind current recommendations. Despite the majority of anaesthesiologists indicating that they follow current fasting guidelines, less than half of them encourage their patients to drink liquids prior to 3 hours to OR, citing variability of OR times, and perception around patient safety, as reasons. However, many anesthesiologists were comfortable with clear fluids up to 4 hours prior to surgery. Given the push for ambulatory surgery, quicker post-operative recovery programs (such as ERAS), and potential detrimental effects of fasting from midnight, we hope our survey will help reveal ways in which traditional fasting policies can be changed to follow more current guidelines. We are investigating by expanding the survey to Europe and Australia/NZ.
References:
Best Pract Res Clin Anesthesiol. 20(3): 363-78, 2006.
Best Pract Res Clin Anesthesiol. 20(3): 483-91, 2006.
